# Endemic Mediterranean seagrasses poised to survive climate change challenges

**DOI:** 10.1002/eap.70235

**Published:** 2026-04-28

**Authors:** Francesco Paolo Mancuso, Mar Bosch‐Belmar, Mario Francesco Tantillo, Martina Russi, Viviana Piermattei, Marco Marcelli, Gianluca Sarà

**Affiliations:** ^1^ Laboratory of Ecology, Department of Earth and Marine Science (DiSTeM) University of Palermo Italy; ^2^ National Biodiversity Future Center (NBFC), Spoke 1 Palermo Italy; ^3^ Consorzio Nazionale Interuniversitario per le Scienze del Mare (CoNISMa) Rome Italy; ^4^ CMCC Foundation ‐ Euro‐Mediterranean Center on Climate Change Lecce Italy; ^5^ Laboratory of Experimental Oceanology and Marine Ecology, Department of Ecological and Biological Sciences (DEB) University of Tuscia, Port of Civitavecchia Rome Italy

**Keywords:** benthic chambers, ecosystem services, net community metabolism, performance curves, *Posidonia oceanica* habitat, prediction maps, species functional traits

## Abstract

Understanding the current and future trajectories of critical habitats is essential for biodiversity conservation and ecosystem management, especially in semi‐enclosed environments such as the Mediterranean Sea. Endemic habitats in the Mediterranean, such as *Posidonia* meadows, are crucial for marine biodiversity, nutrient cycling, oxygen production, and carbon sequestration. Here, using in situ benthic chamber measurements of *Posidonia* meadows integrated with remote sensing data, we developed predictive models of key metabolic traits and upscale their ecosystem service predictions under current and future climate scenarios in the Mediterranean basin. We highlight the essential role of *Posidonia* meadows in providing ecosystem services, such as oxygen production, CO_2_ absorption, and carbon fixation, which are projected to increase, suggesting that *Posidonia* meadows may have some capacity to cope with future ocean warming. However, we also emphasize the importance of other stressors in determining the fate of these key habitats. Our study provides critical insights for guiding coastal management and conservation efforts, contributing to a broader understanding of ecosystem functioning in the Mediterranean Sea. Finally, to illustrate the applicability of our findings, we provide an interactive Shiny app that allows users to spatially explore and estimate the ecosystem services provided by specific *Posidonia* meadows throughout the Mediterranean Sea.

## INTRODUCTION

Marine coastal zones provide essential goods and services crucial for supporting human life on Earth (Barbier et al., 2011; Lopez‐Rivas & Cardenas, 2024). These zones, comprising just 11% of the world's ocean area (Barbier, 2013), support one‐third of the global population and are twice as densely populated as inland areas. Crucial habitats, including seagrass beds, kelp forests, mangroves, salt marshes, and wetlands, flourish in these areas. They offer a broad range of essential provisioning and cultural and regulating ecosystem services (ES) that are vital for the economies and resilience of coastal communities worldwide (Barbier et al., 2011; Cooley et al., 2023; Costanza et al., 1997). The ability of these habitats to provide goods and services that contribute to human well‐being depends directly on their functioning and resilience capability to interacting environmental stressors.

Trait‐based mechanistic approaches in ecology have emerged as a robust framework to predict ecosystem responses to environmental gradients and disturbances, linking biological and ecological traits of species to ecosystem functions, and the resulting ES (McGill et al., 2006; Moreira‐Saporiti et al., 2023). By standardizing the comparison of biodiversity and ecosystem functionality, trait‐based methods facilitate consistent analyses across spatial and temporal scales (Zakharova et al., 2019). In particular, assessing ecosystem traits such as the balance between autotrophic and heterotrophic processes is crucial for understanding the holistic functionality of ecosystems (Meunier et al., 2017). This balance plays a pivotal role in the ecosystem's ability to adapt and respond to environmental changes, offering insights into the dynamics of nutrient cycling and energy flow within ecological communities and their potential role in regulating climate change through the fixation and sequestration of Blue Carbon (Fourqurean et al., 2012; Mazarrasa et al., 2015).

Ocean warming is actively affecting all levels of ecological organization. It is impairing ecosystems and their functioning (Hoegh‐Guldberg & Bruno, 2010), particularly altering metabolic processes of whole communities that are highly temperature‐dependent (Pörtner & Farrell, 2008). Rising temperatures may significantly influence the flux balance of Blue Carbon in coastal marine ecosystems, as ecosystem metabolism can shift towards becoming a source of CO_2_, depending on the metabolic balance of the community (Duarte et al., 2013). With respiration rates tending to increase more rapidly with warming than primary production, warming may shift typically autotrophic ecosystems, such as seagrass meadows, towards net heterotrophic states. Such a shift moves the trophic equilibrium through the transition from CO_2_ sinks to sources, thereby potentially amplifying the effects of local temperature increases due to climate warming (Waycott et al., 2009).

Despite progress in research, accurately valuing and distributing ES remains challenging. For example, the estimate of sanitization role of seagrasses has been done through modeling, and often the output can be validated in the field (Ascioti et al., 2022). Furthermore, predicting how these services will respond to future climate change remains underexplored, limiting our ability to track ecological patterns and implement effective management and policy measures to counteract biodiversity loss and ES degradation (Pecl et al., 2017). Trait‐based methodologies such as those based on oxygen flux changes to measure net community productivity (NCP) have proven essential, although still underused (Mallon et al., 2022), especially in coastal and marine environments where ecosystem degradation significantly impacts the global carbon cycle (Gazeau et al., 2005). Sensor‐based advanced in situ techniques, involving the use of sensorized benthic chambers, have enabled accurate data collection on the metabolic rates of marine communities, critical for conservation efforts aimed at preserving and enhancing “Blue Carbon” storage capacities (Mallon et al., 2022). Such techniques are able to provide precise measurements aiding scientists to monitor ecosystem resilience and environmental stressors, developing strategies for their recovery and preservation.

Seagrass meadows are vital ecosystems performing various ecological functions essential for marine biodiversity enhancement and improving nutrient cycling (Moreira‐Saporiti et al., 2023). It has been estimated that coastal vegetated ecosystems including seagrasses, along with saltmarshes and mangroves, capture up to 70% of the organic carbon buried in the marine sediments, making them some of the most significant carbon sinks of the planet (Duarte et al., 2013). The ES provided by seagrasses are critical to the sustainability of marine life and human economies, and the economic impact of losing these vital habitats has been quantified in thousands of dollars per hectare per year (Costanza et al., 1997; Lopez‐Rivas & Cardenas, 2024). Seagrass habitats are globally declining as a consequence of human activities (Waycott et al., 2009) and the situation is expected to worsen due to climate change (Marín‐Guirao et al., 2018). In the Mediterranean Sea, *Posidonia oceanica* (L.) Delile is among the most representative and functionally performing seagrasses, and its presence is often related to high biodiversity and ecosystem functioning levels (Marbà et al., 2007). The species faces multiple threats, including coastal development, pollution, and the impacts of climate change. Therefore, understanding and predicting changes in ES provided by its meadows is a priority to guide coastal management and restoration actions.

By combining in situ metabolic measurements of the *P. oceanica* community with environmental remote sensing data, we developed predictive models to spatially forecast the distribution of the species' key metabolic traits across the Mediterranean coasts and predict its future changes based on forecasted climate change scenarios. In particular, we used the net community production (NCP) as an important metric to describe organic matter flows in marine communities to investigate the community metabolism of the marine seagrass over yearly temperature conditions, aiming to create ES maps for the habitat under current and future climatic scenarios. Our study seeks to fill critical knowledge gaps essential for predicting the responses of seagrass meadows to environmental changes and for planning conservation efforts. Through this research, we aim to contribute to a broader understanding of how trait‐based mechanistic approaches can be employed to predict ecosystem functioning, particularly in a region as ecologically and economically important as the Mediterranean Sea.

## MATERIALS AND METHODS

This study was performed in the northern Tyrrhenian Sea, in an area off the coast of Civitavecchia (lat 42.076562, long 11.802896, Italy) throughout 2023 (from February to the end of October). The coastal zone hosts areas of high ecological value, including two sites of community importance (SCI; named SCIs IT6000005 and IT6000006) located north and south of the Port of Civitavecchia. These SCIs were established primarily for the protection of marine ecological communities of conservation priority under the European Natura 2000 network, due to the presence of *P. oceanica* meadows and species of conservation priority such as *Corallium rubrum* and *Pinna nobilis* (Bonamano et al., 2021). Studied seagrass meadows were located south of the Port of Civitavecchia at depths ranging from 8 to 10 m, where *P. oceanica* predominantly grows on sandy bottoms formed by the deposition of suspended sediments due to local coastal dynamics (Piazzolla et al., 2024).

### *Posidonia* habitat in situ metabolic measurements

*Posidonia* community metabolism was measured in the field using customized benthic chambers following the method of Roth et al. (2019). The chambers consisted of 10‐mm thick transparent polymethyl methacrylate (PMMA) cylinders (40 cm in diameter and 40 cm in height) with a removable gas‐tender lid (10 mm thickness and 45 cm diameter) that could be easily positioned on the submerged *P. oceanica* community (Appendix S1: Figure S1). The chambers are open at the bottom and are sized to accommodate large portions of the *P. oceanica* community, while still being easily handled by divers. At each site, the benthic chambers were placed haphazardly over *P. oceanica* meadow patches and inserted into the soft sediment ground down of 5 cm. A PVC skirt attached to the chamber's base completely isolated it from the surrounding water environment. Once installed, the chamber encloses a theoretical volume of 44 L (Appendix S1: Figure S1). Each chamber was equipped with sensors for oxygen (Atlas Scientific), temperature (Atlas Scientific), and light (HOBO Pendant MX2202) to measure the O_2_ fluxes in the *Posidonia* community. A magnetic pump facilitated water circulation within the chamber. Sensors were inserted into the lid through a gas‐tight hole. A total of 37 incubations were performed on *P. oceanica* meadows from February to the end of October 2023, encompassing a large range of environmental conditions and seagrass physiological status. Importantly, each incubation captured the metabolic performance of *Posidonia* communities in their natural seasonal state—plants measured at winter temperatures were naturally in winter phenological conditions, while those measured at summer temperatures were in summer conditions. We believe this approach provides a more ecologically realistic thermal performance curve (TPC), as it reflects the response of naturally acclimated communities rather than plants artificially exposed to non‐seasonal temperatures.

*Posidonia oceanica* meadows were incubated for measurements of community respiration (CR) and NCP. CR was measured by covering the chambers with black tissue for 90 min, which was immediately removed to measure NCP during a subsequent 90‐min light incubation. Dissolved oxygen concentration was recorded every 3 s. At the end of in situ incubation, the density of *P. oceanica* and the length of each shoot within the chamber were measured. CR and NCP rates were calculated and expressed as millimoles of dissolved oxygen per square meter per hour per fresh weight (FW) gram (mmol O_2_ g_FW_^–1^ m^2^ h^−1^). Assuming that the hourly rate of CR is constant through the daily cycle, hourly rate of gross primary production (GPP) was estimated as (NCP + |CR| = GCP). Incubations were conducted during mid‐morning to early afternoon hours (approximately 10:00–14:00 local time) when light conditions were relatively stable. GCP was integrated at a daily scale by multiplying the hourly rate by the photoperiod, while daily CR was calculated by multiplying the hourly rate by 24 (Odum, 1956). While we acknowledge that this scaling approach represents a simplification that does not completely account for natural variations in light intensity during dawn and dusk periods, it represents a standard method in benthic metabolism studies (Champenois & Borges, 2021) and provides reasonable first‐order estimates of daily production. Crucially, because our measurements were conducted during the current seasons, photoperiod naturally co‐varied with temperature in an ecologically realistic way—winter measurements occurred under short photoperiods and summer measurements under long photoperiods. Daily NCP, CR, and GCP were visualized according to the season.

Seasonal differences in NCP were assessed using ANOVA. Prior to analysis, normality of residuals was tested using the Shapiro–Wilk test, and homogeneity of variances was assessed using Levene's test. As data violated at least one assumption (*p* < 0.05), we applied the non‐parametric Kruskal–Wallis rank sum test, followed by Dunn's post hoc test with Bonferroni correction for pairwise comparisons. Statistical significance was set at α = 0.05.

### Data analysis

NCP was used to construct the TPC of *P. oceanica* community. NCP was chosen because of its crucial role in ecology, as it represents the balance between the total organic matter produced by photosynthesis (gross community production, GCP) and the total organic matter consumed by respiration (CR). We fitted all 26 nonlinear thermal performance models available in the rTPC package (v. 1.0.2; Padfield et al., 2021) to our temperature–NCP data using nonlinear least squares regression (NLLS). These models represent diverse theoretical frameworks, including mechanistic models based on enzyme kinetics and metabolic theory (e.g., Sharpe–Schoolfield derivatives, Hinshelwood, Pawar), empirical models capturing unimodal thermal responses (e.g., Gaussian, Weibull, Beta, Flinn), and polynomial formulations (e.g., Quadratic) (Padfield et al., 2021). Of the 26 models attempted, 18 converged successfully, while 8 models failed to converge or provide inadequate fits to our data (Appendix S1: Table S1). All models were fitted using the nls.multstart function (Padfield et al., 2021) with multiple starting parameter combinations (iter = 3–6 depending on model complexity) to ensure convergence to global rather than local minima. Model selection was based on the corrected Akaike information criterion (AIC_c_), with models within ΔAIC_c_ ≤ 2 considered to have substantial empirical support (Angilletta, 2006). Once the most accurate model was pinpointed (Appendix S1: Table S1), the model residuals were visually examined using the “test.nlsResiduals” function from the “nlstools” package (Baty et al., 2015), which also conducted formal tests for checking residual normality and spatial–temporal autocorrelation. A bootstrapping method (999 interactions) was employed to resample the data, aiding in the estimation of CIs for model predictions and parameters. Key TPC parameters, including optimal temperature (*T*_opt_), functional thermal range (*T*_fr_), rate at optimum temperature (μ_max_), and optimal thermal breadth (*T*_br‐opt_) were estimated and used to mechanistically illustrate the variations in thermal sensitivities of *P. oceanica* meadows. *T*_fr_ was defined as the temperature range where predicted NCP rates exceeded 20% of μ_max_, representing the thermal window within which *P. oceanica* meadows maintain functional metabolic activity, while the optimal thermal range (*T*_br‐opt_) represents the narrower temperature window where predicted NCP rates exceeded 69% of μ_max_ (Caretto et al., 2015; Matzelle et al., 2015).

### Environmental data

Mediterranean average seasonal sea surface temperature (SST) (Kotsias et al., 2021) raster files were created using daily SST data (Mediterranean Sea Ultra High spatial Resolution [0.01° × 0.01°] SST Analysis) (Buongiorno Nardelli et al., 2013) downloaded from the Copernicus Marine Service Information (https://resources.marine.copernicus.eu). SST layers were downloaded for the three years preceding the field experiment (2021–2023). Forecasted daily SSTs for the year 2050 were obtained from pre‐processed model outputs produced under representative concentration pathways (RCP) 4.5 and 8.5 (IPCC, 2014). These scenarios were selected to capture a moderate and an extreme trajectory of future climate change. RCP 4.5 represents a likely range of future global moderate greenhouse gas (GHG) and aerosol emissions, whereas RCP 8.5 represents a high‐emission, worst case scenario included for comparative purposes. The dataset consisted of downscaled, bias‐corrected projections at daily resolution, provided as ready‐to‐use NetCDF files. All SST raster layers were upscaled to a resolution of 0.08° × 0.08° decimal degrees. Daily SST data were grouped by season following the definitions provided by Kotsias et al. (2021): winter (3/12–22/3), spring (23/3–14/6), summer (15/6–5/9), and autumn (6/9–2/12), as day/month format. Then, average raster per season was made and used in the following analysis.

Spatial and temporal predictions of *P. oceanica* meadows NCP were calculated by integrating NCP measurements and the seasonal present and projected SST layers with a georeferenced habitat distribution layer provided by EMODnet Seabed Habitats (Vasquez et al., 2023).

### Trait‐based ecosystem functioning maps

The model best fitting NCP data was used to spatially predict (at pixel level) the potential community metabolism (NCP) of *P. oceanica* habitat in the present‐day climatic conditions and in 2050 future scenarios of GHG emissions (RCP 4.5 and 8.5) (Thomson et al., 2011).

To spatially visualize patterns in metabolic performance, we categorized thermal performance into three zones: optimal performance range (>69% of μ_max_), suboptimal performance range (20%–69% of μ_max_), and pessimum performance range (<20% of μ_max_) (Bosch‐Belmar et al., 2022).

To facilitate visualization of maps, NCP values predicted by the model in all scenarios were categorized in three different groups: pixels containing NCP values within the range identified by the *T*_br‐opt_ of the species (>69% of μ_max_) were colored green; those with values within the suboptimal performance range (20%–69% of μ_max_) were colored orange; and pixels with metabolic values within the pessimum performance range (<20% of μ_max_) were colored red. Temporal and spatial differences between present and future predictions for the species' performance were visualized by performing subtractions between future and present rasters. The values of the newly obtained raster layers represented the predicted increase or decrease of metabolic performance of the habitat at pixel level. In addition, low and upper thermal‐safety margins were calculated (Sunday et al., 2014) through the offset between CT_min_ and CT_max_ and minimum and maximum environmental temperature experienced within the Mediterranean basin over the year. Finally, the spatial and temporal metabolic performances of the *P. oceanica* habitat were expressed as the total million tons (Mt) of O_2_ released, CO_2_ absorbed and C fixed per season, year, and across the three Mediterranean sectors (west, center, east) according to Dimarchopoulou et al. (2021). In doing so, we assumed that, since NCP measures the net amount of carbon fixed by the plants that contributes to their growth and biomass after respiration, it directly relates to the process of carbon fixation. Oxygen production was converted into CO_2_ absorption and C fixation by assuming a theoretical molar ratio of CO_2_:O_2_ = 1, C:O_2_ = 1 (Roth et al., 2019).

## RESULTS

**FIGURE 1:**
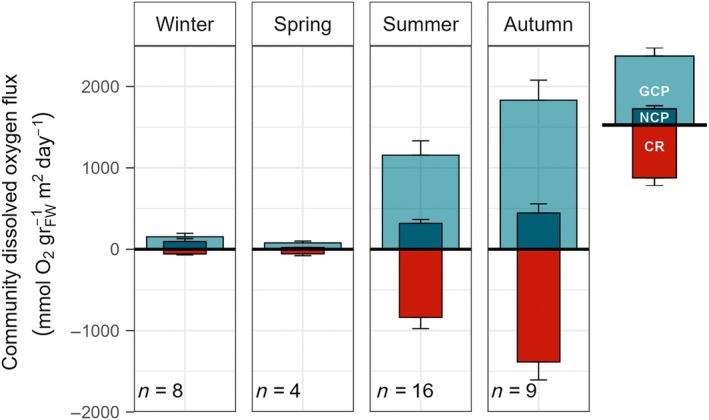
Daily *Posidonia* community dissolved oxygen fluxes (in millimoles of dissolved oxygen per square meter per hour per fresh weight) from in situ benthic chamber incubations. CR, community respiration; GCP, gross community production; NCP, net community production. Values are given as mean ± SE.

The daily NCP and CR of *P. oceanica* ranged from 94.8 ± 34.6 to 446.0 ± 111.0 mmol O_2_ g^−1^_FW_ m^−2^ day^−1^ and from 58.0 ± 11.5 to 1385.0 ± 221.0 mmol O_2_ g^−1^_FW_ m^−2^ day^−1^, respectively; while the daily GCP ranged from 153.0 ± 43.3 to 1831.0 ± 247.0 mmol O_2_ g^−1^_FW_ m^−2^ day^−1^ (Figure 1). The range of NCP values computed indicate that *P. oceanica* habitats exhibit autotrophic metabolism throughout the year. Significant seasonal differences were observed (Kruskal–Wallis: χ^2^ = 16.23, df = 3, *p* = 0.00102; Appendix S1: Table S1, Figure S2). Post hoc pairwise comparisons (Dunn's test with Bonferroni correction) revealed that spring exhibited the lowest NCP values (21.3 ± 2.7 mmol O_2_ g_FW_^−1^ m^−2^ day^−1^, *n* = 4), significantly lower than that of both autumn (446.0 ± 111.2 mmol O_2_ g_FW_^−1^ m^−2^ day^−1^, *n* = 9; *p* = 0.007) and summer (318.5 ± 48.0 mmol O_2_ g_FW_^−1^ m^−2^ day^−1^, *n* = 16; *p* = 0.016). Autumn NCP was also significantly higher than that of winter (94.8 ± 34.6 mmol O_2_ g_FW_^−1^ m^−2^ day^−1^, *n* = 8; *p* = 0.042; Appendix S1: Table S1, Figure S2).

**FIGURE 2:**
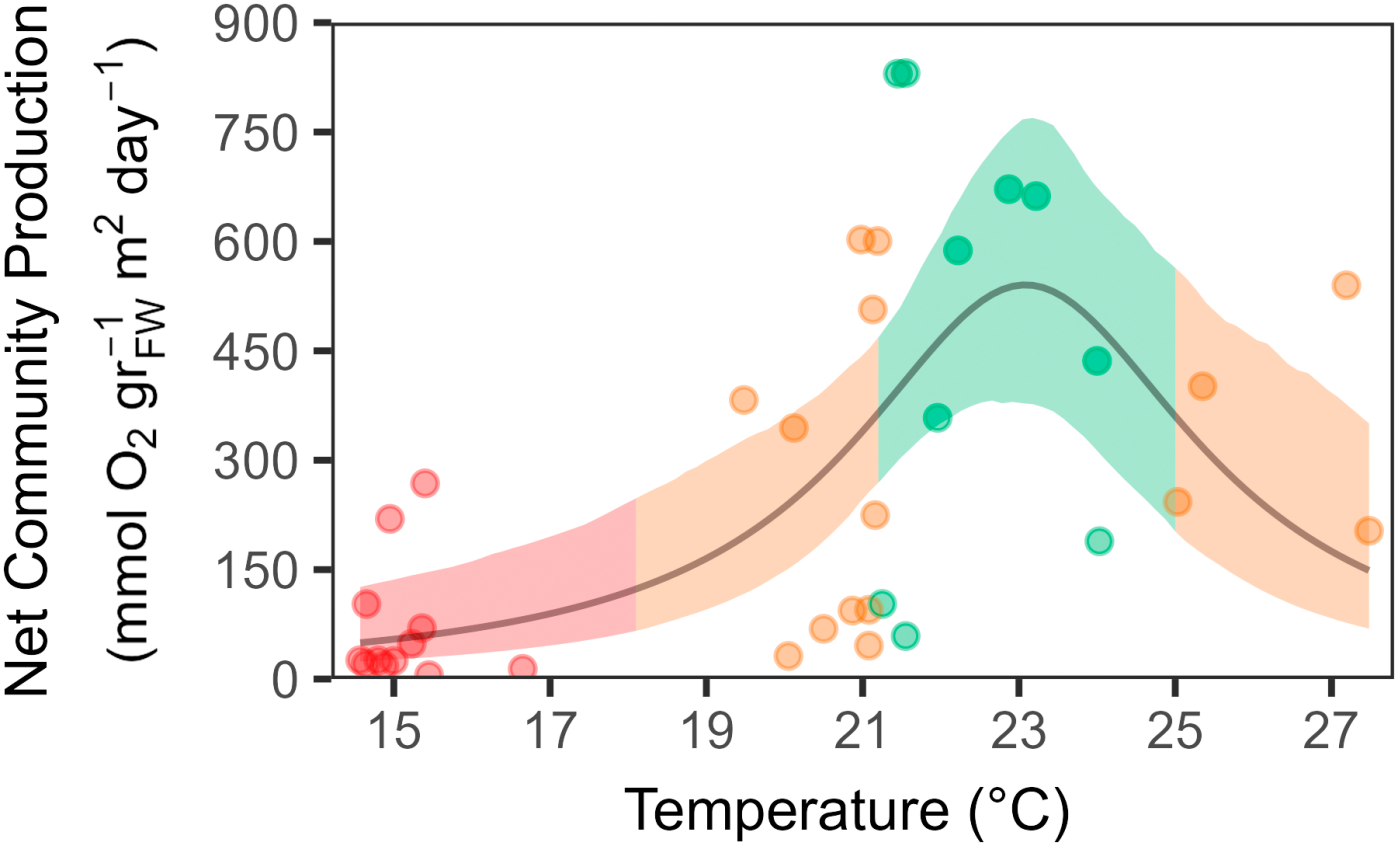
Net community production (NCP) of *Posidonia oceanica* habitat. Thermal performance curve of *P. oceanica* community in relationship to in situ temperature conditions over the year. Solid lines represent the average fitted values according to the selected model (*Flinn*). Shaded area is bootstrapped prediction showing ± 95% CI based on 999 iterations. The different colors represent optimum thermal breath (*T*_br‐opt_) (green colored), suboptimal range (orange), and pessimum thermal range (red).

Among the 26 available models present in the rTPC package, two models met the ΔAIC_c_ ≤ 2 criterion: Flinn (1991) with the best fitting (AIC_c_ = 505.89, ΔAIC_c_ = 0, weight = 0.305) and Lynch and Gabriel (1987) and Vasquez et al. (2023) as a competitive alternative (AIC_c_ = 506.96, ΔAIC_c_ = 1.07, weight = 0.179). The third‐best model (Quadratic) had ΔAIC_c_ = 2.52, falling outside the threshold (Appendix S1: Table S2). Given the substantially higher Akaike weight of the Flinn model (1.7× greater than Gaussian), we selected it as the primary model for constructing the TPC of *P. oceanica*. The three fitted coefficients calculated by the model were used to generate the TPC curve (Figure 2, Appendix S1: Table S3). The curve presented a slightly left‐skewed form with a thermal optimum (*T*_opt_) around 23°C, where the species exhibits maximum performance. Species' thermal tolerance presented a range of 24°C, with minimum and maximum critical temperatures (CT_min_ and CT_max_) identified at 11 and 35°C, respectively (Appendix S1: Table S3). Optimal thermal tolerance windows and functional thermal range included temperature ranges of 3.82 and 9.93°C, respectively (Appendix S1: Table S3). The habitat presented a positive upper thermal‐safety margin of 6.1°C and a negative minimum thermal‐safety margin of 2.1°C.

**FIGURE 3:**
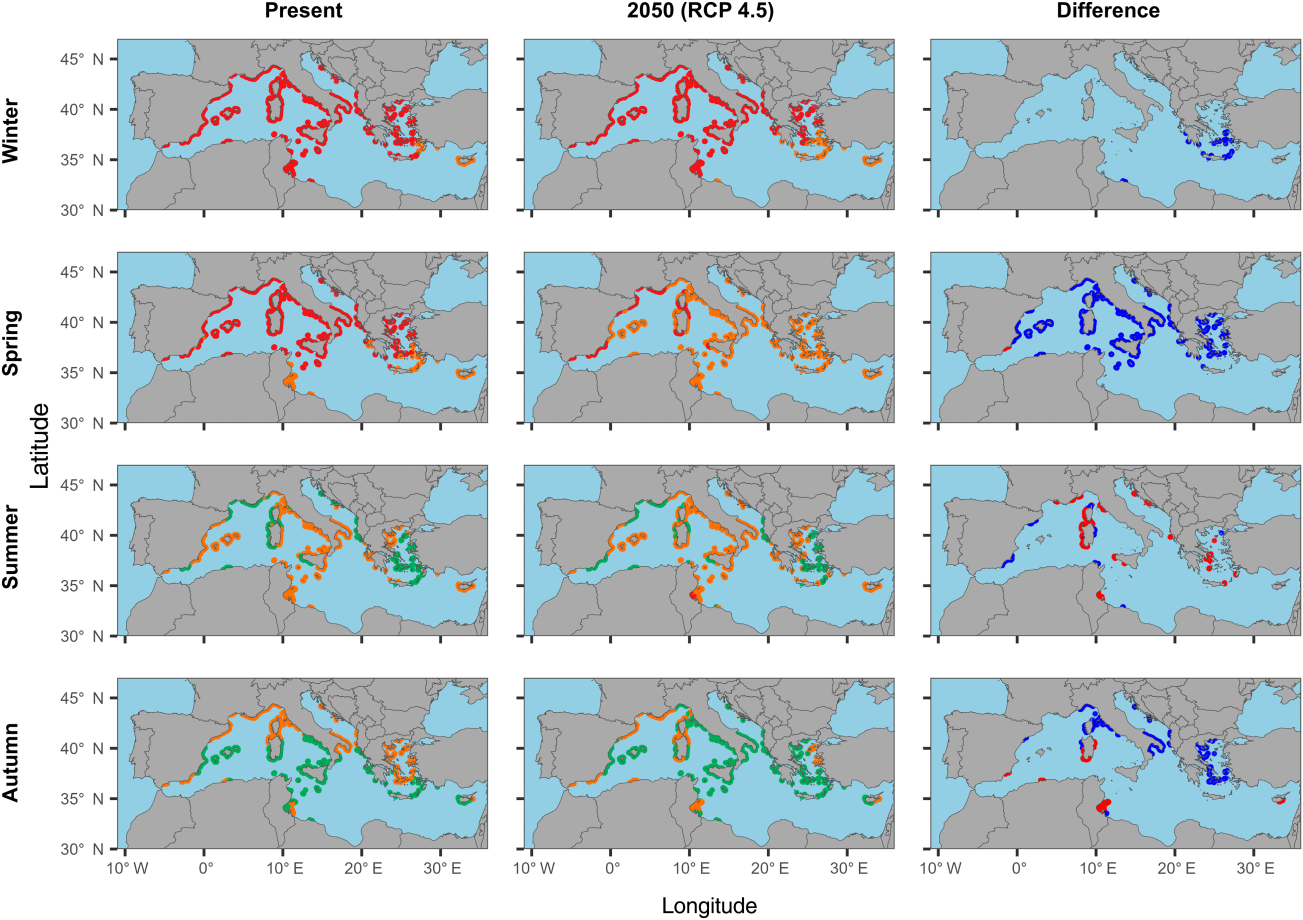
Spatiotemporal present (left column) and future 2050 (RCP 4.5, central column) predictions of the metabolic performance (NCP) of *Posidonia oceanica* habitat along the Mediterranean coasts. The different colors represent optimum thermal breath (*T*_br‐opt_) (green colored), suboptimal range (orange), and pessimum thermal range (red) as reported in Figure 2. The right column shows differences between future and present predictions. In this column, red color indicates a decrease in the metabolic performance of the *Posidonia* habitat, while blue color highlights an increase in metabolic performance.

The estimated total area covered by the *Posidonia oceanica* habitat in the Mediterranean is 1.31 million hectares (Mha), with the largest extent occurring in the center (646,500 ha) and western (606,600 ha) sectors, and the smallest in the eastern part of the basin (45,900 ha). Predictions from the present spatiotemporal analysis of the NCP of *Posidonia* showed variations across seasons and geographical locations. In winter and spring, a larger part of *Posidonia* meadows exhibited metabolic performance outside the predicted *T*_fr_ of the species (Figure 3, red areas). Only a few small areas, mainly in Tunisian and Greek regions, showed suboptimal performance (Figure 3, orange areas). In summer and autumn, the metabolic performance of the *Posidonia* habitat increased, presenting more abundant highly performant meadows (with optimum NCP performance, green areas) in autumn compared to summer (Figure 3).

Predicted future NCP rates (2050, RCP 4.5) for *P. oceanica* showed significant changes in spring, with a large part of the meadows increasing their metabolic performance (Figure 3). On the contrary, predictions showed a decrease in NCP in different *P. oceanica* meadows during summer months, especially along the eastern coasts of Corsica, Sardinia, and Sicily, although locally there is also an increase in performance in some west coast areas (Figure 3). Finally, in autumn, there was an increase in NCP especially along the coasts of Corsica, Apulia, and Greece, while there was a decrease in performance along the central and southern coasts of Sardinia and a large part of the Tunisian meadows (Figure 3). Overall, autumn appeared to be the season with the highest metabolic performance, when seawater temperatures remain within the optimal range for *P. oceanica* meadows.

**FIGURE 4:**
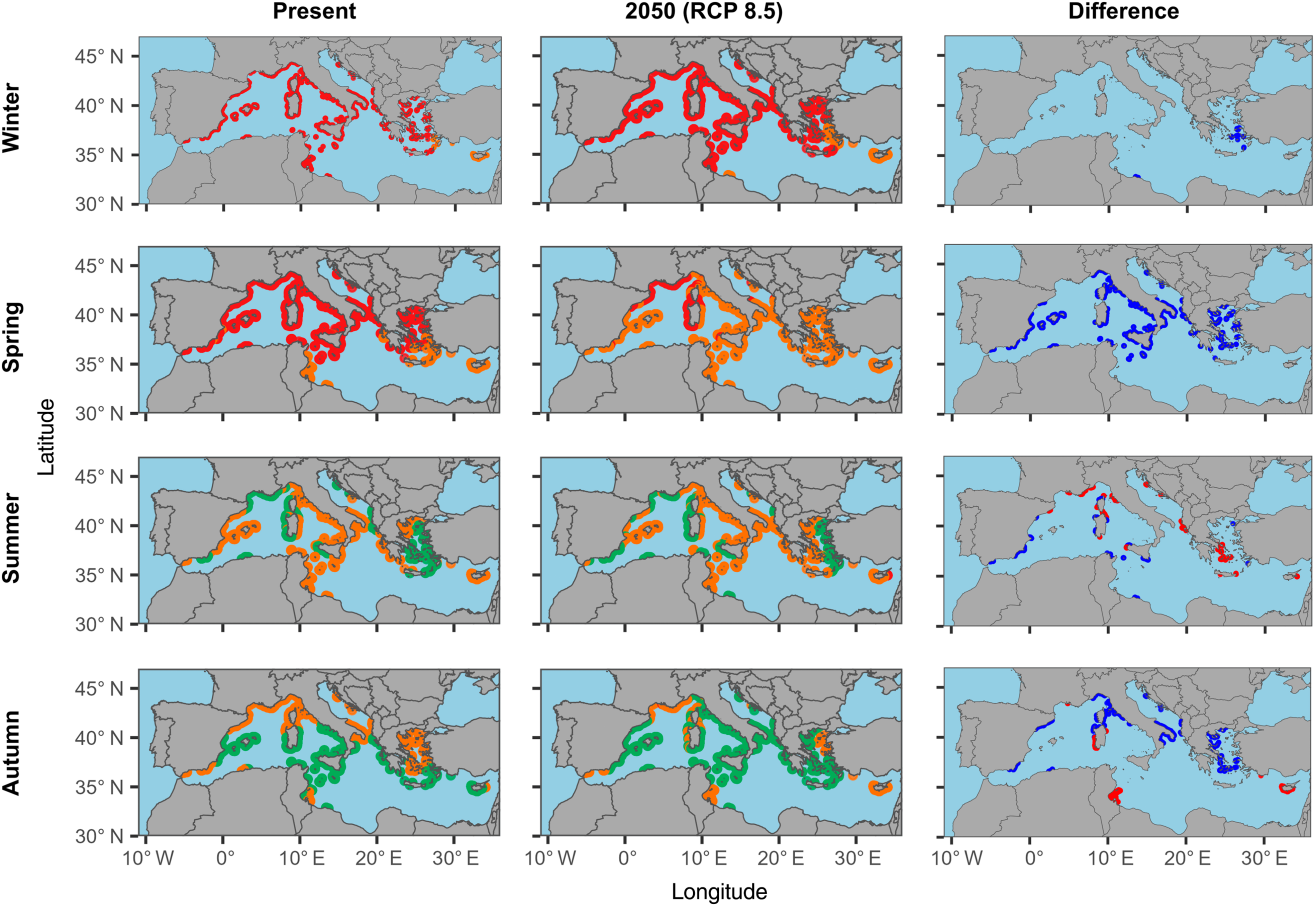
Spatiotemporal present (left column) and future 2050 (RCP 8.5, central column) predictions of the metabolic performance (NCP) of *Posidonia oceanica* habitat along the Mediterranean coasts. The different colors represent optimum thermal breath (*T*_br‐opt_) (green colored), suboptimal range (orange), and pessimum thermal range (red) as reported in Figure 2. The right column shows differences between future and present predictions. In this column, red color indicates a decrease in the metabolic performance of the *Posidonia* habitat, while blue color highlights an increase in metabolic performance.

Predicted future NCP rates for *P. oceanica* under the RCP 8.5 scenario showed a broadly similar pattern to RCP 4.5, with an overall increase in metabolic performance during summer and autumn and a more variable response across the rest of the year (Figure 4). Increased NCP was observed in the Alboran Sea (Strait of Gibraltar), where present‐day cooler waters are expected to reach temperatures closer to the species' optimum under the stronger warming signal of RCP 8.5. During summer, improved performance was also evident in the southern Mediterranean, particularly along the western coasts of Corsica and Sardinia, southern Sicily, and the Tunisian coastline. In contrast, autumn projections resembled those under RCP 4.5, with higher NCP along much of the western basin but a more pronounced decline in the eastern Mediterranean, especially around Cyprus. Overall, as in the present‐day and RCP 4.5 conditions, the highest metabolic performance was predicted for autumn, when seawater temperatures are generally closer to the thermal optimum for *P. oceanica* meadows.

In addition, to explore the potential physiological range of *P. oceanica* beyond its current distribution (i.e., the species' fundamental thermal niche), we also generated present‐day and RCP 4.5–8.5 NCP maps without applying the EMODnet habitat mask (Appendix S1: Figures S3 and S4). These predictions highlight areas that could be suitable for meadow establishment under current and moderate future temperature regimes, particularly along the southern Mediterranean coasts, where ecological information is still scarce.

**FIGURE 5:**
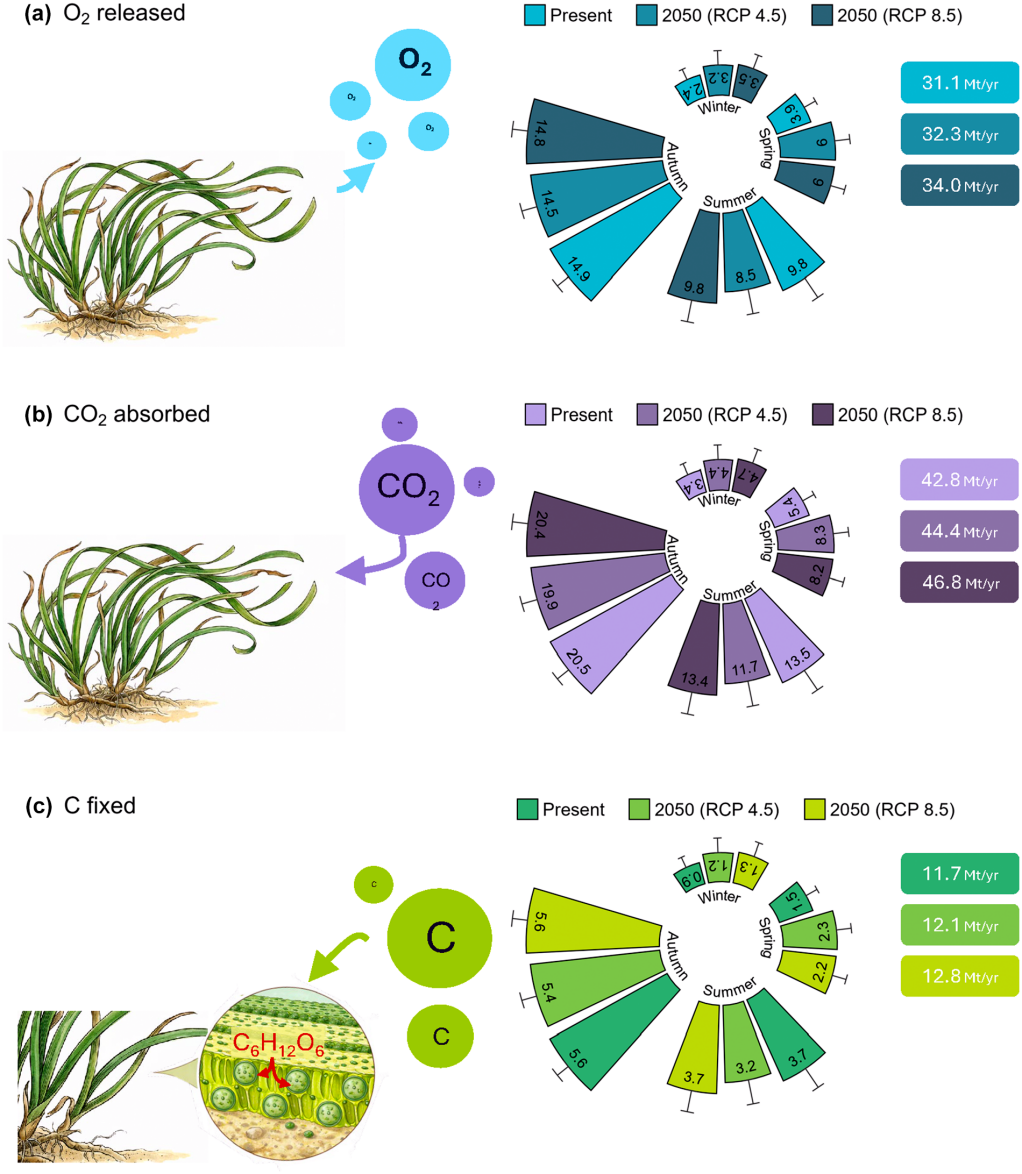
Total million tons of O_2_ released (a), CO_2_ absorbed (b), and C fixed (c) by *Posidonia oceanica* habitat per season and year for the present and projected 2050 (RCP 4.5 and RCP 8.5) seawater temperatures. Bars show mean + SE. See Appendix S1: Figure S5 for data concerning the main three Mediterranean sectors (west, center, east). Illustrations by Francesco Paolo Mancuso.

The translation of the present predicted spatiotemporal variation of NCP into the amount of O_2_ produced, CO_2_ absorbed, and C fixed by *Posidonia* habitat revealed that the total amount of oxygen produced in the Mediterranean Sea varied seasonally, ranging from 2.4 Mt in winter to 3.9 Mt in spring. This amount more than doubled in summer and autumn periods, reaching 9.8 and 14.9 Mt, respectively. This adds up to a total of 31.2 Mt year^−1^ O_2_ produced (Figure 5). Values of absorbed CO_2_ and C fixed followed the same pattern, with the lowest values in winter (CO_2_ = 3.3 Mt, C = 0.9 Mt) and spring (CO_2_ = 5.4 Mt, C = 1.5 Mt), and higher values in summer (CO_2_ = 13.5 Mt, C = 3.7 Mt) and autumn (CO_2_ = 20.5 Mt, C = 5.6 Mt), corresponding to a total of 42.8 and 11.7 Mt year^−1^, respectively, for CO_2_ and C (Figure 5). Under the RCP 4.5 scenario, O_2_ production, CO_2_ absorption, and C fixation were predicted to increase by 33.3% in winter and 53.8% in spring, while decreasing by 13.8% in summer and 2.7% in autumn (Figure 5). This results in an overall amount of O_2_ produced, CO_2_ absorbed, and C fixed for future *Posidonia* habitat in the Mediterranean Sea of 32.3, 44.4 and 12.1 Mt year^−1^, respectively (Figure 5). When compared with RCP 4.5, projections under the RCP 8.5 scenario showed a broadly similar seasonal pattern but with slightly higher values overall (Figure 5). Oxygen production increased during winter and spring, following the same trend observed under the moderate emission scenario, whereas the seasonal reductions in summer and autumn were less pronounced. The total amount of O_2_ produced across the Mediterranean basin was estimated at 34.0 Mt year^−1^, representing a 5.3% increase compared with RCP 4.5. Similarly, total CO_2_ absorption and C fixation reached 46.8 and 12.75 Mt year^−1^, corresponding to increases of 4.7% and 5.4%, respectively. Overall, RCP 8.5 projections suggest a modest basin‐wide enhancement of ES, mainly driven by higher winter and spring productivity (Figure 5).

## DISCUSSION

Predictions based on in situ benthic chamber up‐scaling of *P. oceanica* habitat metabolic traits confirm the relevant role that this seagrass plays in providing ES related to water column oxygenation (Agueda Aramburu et al., 2024), as well as CO_2_ absorption and carbon fixation (Pergent‐Martini et al., 2021; Traganos et al., 2022) at the Mediterranean scale. Rising temperatures forecasted under climate change scenarios may enhance the ability of the seagrass to provide vital ES, underscoring the critical importance of conserving *Posidonia* habitats to safeguard the health of future ecosystems. *P. oceanica* meadows support a broad range of metabolic rates and tend to be autotrophic systems throughout the year, acting as one of the most intense carbon sinks on the planet (Macreadie et al., 2014) comparable to peatlands and mangrove systems (Pergent‐Martini et al., 2021).

In situ metabolic measurements of daily NCP, CR, and GCP align with those previously reported in the literature (Bosch‐Belmar et al., 2025; Champenois & Borges, 2021) in terms of both yearly variation and average daily metabolic performance, and allowed us to estimate a seasonal daily mean NCP ranging from 21 to 446 mmol O_2_ m^–2^ day^–1^, depending on the season. Such community metabolism values fall within the range calculated by Duarte et al. (2010) for seagrasses worldwide and are also similar to the NCP values obtained for different *P. oceanica* meadows in France (37.21–123 mmol O_2_ m^−2^ day^−1^; Champenois & Borges, 2021), Italy (54–119 mmol O_2_ m^−2^ day^−1^; Koopmans et al., 2020), and Spain (NCP measured values up to 98 mmol O_2_ m^−2^ day^−1^; Gazeau et al., 2005).

For the first time, field‐measured community metabolism (NCP) over seasonal temperature variations was used to build a reliable TPC for the habitat. Our approach of measuring metabolism in situ across different seasons captures the ecologically real thermal niche of *Posidonia* communities—how meadows actually perform when naturally acclimated to different temperatures and phenological states throughout the year. Importantly, this field‐based approach is strongly validated by our independent mesocosm study (Bosch‐Belmar et al., 2025), which tested metabolic responses of seasonally acclimated plants to controlled temperature variations (ambient, +2°C, +4°C) within three seasonal periods. Despite the different methodological approaches and ecological scales (community vs. individual), both studies identified the same thermal optimum (*T*_opt_ = 23°C), confirming that our field‐derived TPC accurately captures the species' thermal response rather than spurious correlations with co‐varying seasonal factors, (Bosch‐Belmar et al., 2025), underscoring the plant's predominant role in community metabolism. While our field‐based TPC integrates multiple naturally co‐varying environmental factors (temperature, photoperiod, phenology, nutrient dynamics, and biogeochemical conditions), we believe this integrated response is precisely what is needed for predicting ES under future climate scenarios, as these factors will continue to co‐vary with temperature under climate change. The previously mentioned mesocosm study (Bosch‐Belmar et al., 2025) allowed disentangling some of these effects under controlled conditions. In those experiments, photoperiod and light intensity were specifically controlled according to season characteristics (50, 80, and 92 μmol photons m^−2^ s^−1^ for winter, summer, and autumn, respectively, matching natural conditions at 10 m depth), eliminating concerns about photoperiod scaling effects on daily production estimates. Mechanistic indicators including maximum electron transport rate (ETR_max_) through rapid light curves and chlorophyll fluorescence analysis were also measured. ETR_max_ showed the same thermal optimum (~23°C) as our field‐measured NCP, confirming that temperature effect on photosystem II electron transport is the primary driver of the thermal performance patterns, rather than artifacts of light‐temperature co‐variation. Additionally, the mesocosm study separately analyzed net photosynthesis (*P*_*n*_), dark respiration (*R*_*d*_), and net primary production (NPP) across the same temperature range. While respiration showed a more linear increase with temperature up to 31°C without a clear optimum (consistent with the metabolic theory of ecology and general biochemical kinetics), both *P*_*n*_ and NPP exhibited clear thermal optima at 23°C, similar to our field‐measured NCP. This indicates that the autotrophic photosynthetic component dominates the thermal response of NCP in *P. oceanica* meadows, although we acknowledge that heterotrophic components (e.g., microbial communities, meiofauna) may exhibit different thermal sensitivities. Future research explicitly quantifying the separate thermal responses of autotrophic versus heterotrophic components within *Posidonia* meadows, potentially through stable isotope approaches or selective inhibitor studies, could further refine predictions of community metabolism under warming scenarios and identify potential shifts in the balance between these components.

Recent studies indicate that the optimal temperature for the species is around 26°C, while the upper critical temperature (CT_max_) is 35.5°C (Rinaldi et al., 2023; Savva et al., 2018). The differences in *T*_opt_ are likely due to the fact that our measurements were taken on meadows located at 10 m depth, whereas previous studies were conducted at shallower depths (i.e., 1–5 m; see Savva et al., 2018). In contrast, similar CT_max_ values were observed, with previous studies reporting 35.5°C, which is remarkably consistent with our estimate (35.1°C), confirming the robustness of this upper thermal limit for the species. In addition, the thermal safety margin identified in our field data (6.1°C) indicates that the habitat may have sufficient thermal tolerance to cope with extreme warm temperatures.

Upscaling metabolic measurements collected through benthic chambers allowed us to obtain an overall, although approximated, view of the metabolic performances of *Posidonia* habitats at the Mediterranean scale. We acknowledge that our field‐based approach captures the integrated response of naturally co‐varying environmental factors rather than isolating the independent effect of temperature alone. However, the validation of our thermal optimum through controlled mesocosm experiments (Bosch‐Belmar et al., 2025), where photoperiod, light intensity, turbidity, and other factors were systematically controlled while temperature was manipulated within seasonally acclimated plants, provides strong evidence that the thermal optima and thresholds we identified represent fundamental physiological properties of *P. oceanica* meadows rather than statistical artifacts of seasonal correlations. Our study site in the Tyrrhenian Sea represents mid‐depth (8–10 m) meadows in the central Mediterranean under typical oceanographic conditions for this region. While this site provides valuable insights into the metabolism and thermal responses of *Posidonia*, extrapolating locally derived relationships to the entire Mediterranean basin may introduce spatial uncertainty. In particular, our approach assumes that thermal physiology is broadly consistent among Mediterranean *Posidonia* populations and that the TPC derived from a central Mediterranean site can be generalized across the species' range. Although population genetic studies reveal both historical connectivity and regional genetic structure across *P. oceanica* meadows (Arnaud‐Haond et al., 2007; Serra et al., 2010), the species also exhibits considerable phenotypic plasticity (Moreira‐Saporiti et al., 2023; Serra et al., 2010). The independent validation of our thermal optimum strongly indicates that the fitted curve represents the central tendency of the species' community‐level thermal response (Bosch‐Belmar et al., 2025). Nevertheless, populations experiencing divergent temperature regimes over evolutionary timescales (e.g., warmer eastern Mediterranean or cooler northern Adriatic) may deviate from this pattern. Therefore, our models should be interpreted as a first‐order mechanistic framework that provides a foundation for Mediterranean‐scale metabolic projections, which could be refined through future comparative studies across genetically distinct populations, identifying potential hotspots of local thermal adaptation.

Our modeling approach shows that O_2_ production, CO_2_ absorption, and C fixation ability are likely to change in the future. *P. oceanica* populations currently present in the Mediterranean will experience a temperature‐dependent net increase in performance, particularly in spring when higher temperatures will favor greater NCP in almost all the meadows. Although from a purely numerical standpoint, this aspect seems to benefit the functioning of future Mediterranean marine ecosystems, it is important to note that our model mainly accounts for direct effects of temperature on community metabolism. While our predictions suggest temperature‐dependent increases in metabolic performance, other aspects of *P. oceanica* ecology remain uncertain and could shape future outcomes. For example, future temperature changes may modify the species' phenology and sexual reproduction events. Indeed, plant flowering is often affected by climate change, both in terms of timing and intensity, potentially affecting reproductive phenology. Phenological modifications are among the most important mechanisms influencing changes in plant population distribution and abundance over both short and long term (Diaz‐Almela et al., 2007). Therefore, while our metabolic predictions indicate resilience to moderate warming, the full response of *Posidonia* meadows to climate change involves complex interactions that introduce additional uncertainty beyond the scope of our current model.

Despite the overall increase in performance, a decrease in the *P. oceanica* community's performance during the warmest period was observed in the RCP 4.5 scenario predictions, especially in meadows located in the central‐western Mediterranean (e.g., around Sardinia and western Sicily). Then, future high temperatures in this area could push the meadows to stressful thermal conditions, potentially causing a metabolic collapse due to photosystems malfunctioning. This metabolic deterioration could reduce the habitat quality and ES that *Posidonia* meadows provide, including their role as nursery grounds and feeding areas for commercially important fish species. Such degradation of these critical habitats would consequently impair local coastal fisheries' landings and income (El Zrelli et al., 2020).

Even though water warming does not appear to be a limiting factor for the functioning and capacity of the habitat to provide provisioning and regulating ES, other sources of disturbance related to climate change such as sea level rise and increased wave force have been suggested as potentially detrimental to the habitat (Boudouresque et al., 2009; Infantes et al., 2009; Pergent et al., 2015). The impact of extreme climatic events such as marine heatwaves (MHWs) is an important factor potentially influencing community structure and functioning, above all impairing performances of foundation species (Sarà et al., 2021); nonetheless, integrating these events into modeling predictions is currently challenging. MHWs have increased in frequency, intensity, and duration in recent years, subjecting many marine communities, especially benthic ones, to intense thermal stress and mass mortalities (Garrabou et al., 2022). In addition, the cumulative impact of different anthropogenic stressors may cause a regression in the distribution and extent of *P. oceanica* habitat. Several studies have identified a vast variety of pressures as causes of *P. oceanica* meadows declines, being eutrophication (i.e., urban and industrial sewage and aquaculture activity), coastal modification due to dredge/fill activities (Aragonés et al., 2015; Badalamenti et al., 2011; Manzanera et al., 2011), bottom trawling and anchoring boating (Pergent‐Martini et al., 2022), and natural stressors such as biological invasions and erosion due to increasing wave energy (Infantes et al., 2009; Marbà et al., 2014) among the most cited and studied stressors impacting *P. oceanica* distribution. Conversely, some studies suggest that this crucial habitat for Mediterranean biodiversity might not be in decline. For example, Bonacorsi et al. (2013) stated that the extent of *P. oceanica* meadows has remained stable along 50 years and that the observed differences may be due to significant improvements in the technologies used for monitoring the species' distribution. Overall, this evidence suggests that while future increases in water temperatures may not inherently limit the metabolic performance and ES provided by *Posidonia*, the increasing number of interacting stressors, especially near urban areas, will continue to pose significant uncertainty regarding the future persistence of *Posidonia* habitats.

Seagrass meadows are habitats of intense metabolism, supporting both high GCP and CR, which are often closely balanced (Larkum et al., 2006). The community metabolic balance will determine whether a community exports organic carbon to adjacent systems (net autotrophic; NCP > 0) or whether a community requires external organic carbon inputs to sustain its own metabolism (net heterotrophic; NCP < 0). Current assessments of seagrass carbon budgets exhibit considerable variability and fluctuate seasonally. For instance, some studies report that *Posidonia* seagrass communities may act as carbon sources during winter and as carbon sinks throughout the rest of the year (Macreadie et al., 2014). In contrast, other studies have found that *Posidonia* meadows show autotrophic metabolism round the year (Agueda Aramburu et al., 2024; Champenois & Borges, 2021). The meadows studied here showed net autotrophic metabolism throughout the year across the Mediterranean, functioning as a carbon sink with remarkable seasonal differences. This highlights the vital importance of these meadows in maintaining ecological balance, supporting marine life, and combating global environmental challenges due to human‐caused stressors such as climate change and side effects.

The surface area covered by the habitat of *P. oceanica* meadows in the Mediterranean Sea has been the subject of several estimates in the past (Pergent‐Martini et al., 2021; Telesca et al., 2015; Traganos et al., 2022), highlighting the scarcity of data for the eastern basin and along the African coasts. The lack of information or the absence of the seagrass habitat in certain areas of the Mediterranean does not necessarily mean that the species is not present or cannot be present. By using the same approach applied in this study, without restricting the predictions to the areas where the habitat is currently present, it is possible to identify the fundamental thermal niche of the habitat—that is, the maximum area *Posidonia* could potentially occupy as a function of temperature. This analysis shows that, in particular, the southern and eastern coasts of the Mediterranean emerge as areas where the habitat, if present, could exhibit high functionality, both now and in the future, significantly further contributing to the ES provided by seagrasses (Appendix S1: Figure S5). However, given the importance of these habitats for the overall carbon exchanges in the Mediterranean, this bias needs to be addressed. A detailed georeferenced distribution map of *Posidonia* habitats suggests that our predictions may underestimate the ES provided by this seagrass.

Although carbon sink estimation by *P. oceanica* meadows is usually based on tissue carbon content analysis, to our knowledge, this is the first attempt to use metabolic traits to spatially and temporally represent the community functionality of *P. oceanica* habitats. Estimations calculated from in situ metabolic community functions (NCP) showed that the seagrass habitat in the Mediterranean is able to produce 31 Tg O₂ yr⁻¹ O_2_ per year, absorb 43 Tg O₂ yr⁻¹ CO_2_ per year, and fix 12 Tg O₂ yr⁻¹ C per year for an estimated area of 1.3 million ha. Future projections suggest that warming will not uniformly reduce ecosystem functioning across the basin. Moderate warming may initially enhance metabolic activity in cooler areas of the Mediterranean, while extreme warming could still sustain net autotrophy in many regions, especially during the cooler seasons when temperatures approach the species' thermal optimum.

In conclusion, this study underscores the critical role of *P. oceanica* meadows in Mediterranean ecosystems, particularly in light of ongoing and projected climate change. Through in situ metabolic measurements using benthic chambers, we confirmed the seagrass habitats' significant contribution to ES such as water column oxygenation, CO_2_ absorption, and carbon fixation. The metabolic traits observed reflect robust community performance across diverse Mediterranean locations, with implications for regional carbon budgets and ecosystem stability. Importantly, our findings suggest that future warming scenarios may enhance these services, particularly during autumn when optimal temperatures promote higher NCP. However, challenges remain, as elevated temperatures could also lead to seasonal declines in performance, especially in central‐western Mediterranean regions vulnerable to thermal stress. Given these complexities and the spatial uncertainties inherent in extrapolating locally derived TPCs across the Mediterranean basin, conservation efforts must be adaptive and informed by the best available scientific tools.

From an applied perspective, these findings provide a valuable baseline for climate‐adaptive management of Mediterranean seagrass meadows. Identifying areas projected to maintain positive metabolic performance under future climatic scenarios can help define potential thermal refugia, where conservation and restoration efforts may be most effective. Conversely, regions expected to experience greater metabolic decline should be prioritized for mitigation strategies aimed at reducing cumulative stressors such as eutrophication, mechanical disturbance, and coastal pollution. To facilitate the practical application of our findings, we developed an interactive Shiny application (https://seadataweb.shinyapps.io/Posidonia_ES_map/) to translate our findings into a practical decision‐support platform. This application should be viewed as an exploratory platform for visualizing first‐order spatial trends in *Posidonia* ecosystem functioning rather than a deterministic forecasting model. Its primary purpose is to assist coastal managers and policymakers in identifying areas potentially sensitive to temperature changes and in prioritizing locations for conservation interventions or more detailed local assessments. The application allows users to explore spatially explicit estimates of ES (oxygen production, CO_2_ absorption, and carbon fixation) across Mediterranean *Posidonia* meadows under current and future climate scenarios. Users are encouraged to interpret local results in light of site‐specific environmental conditions, recognizing that our basin‐scale model represents a first‐order approximation based on central Mediterranean thermal physiology. The tool facilitates exploration of the underlying data and spatial variability in predicted ES, while always acknowledging that local results must consider possible site‐specific adaptations, depth‐related differences, and environmental conditions not captured in our simplified thermal model. In this way, the application provides a scientifically grounded yet flexible baseline to inform future research directions and conservation strategies across the Mediterranean basin. Integrating such high‐resolution temporal and spatial information into broader ecosystem management strategies will be essential for mitigating the impacts of climate change and other anthropogenic stressors. By combining mechanistic trait‐based modeling with user‐friendly visualization tools, we aim to bridge the gap between scientific research and practical conservation action, enabling practitioners to make more informed decisions about the protection and restoration of these critical coastal ecosystems.

## AUTHOR CONTRIBUTIONS

Francesco Paolo Mancuso, Mar Bosch‐Belmar, and Gianluca Sarà: idea conception and experimental design. Francesco Paolo Mancuso, Mar Bosch‐Belmar, Mario Francesco Tantillo, and Martina Russi: data acquisition. Francesco Paolo Mancuso and Mar Bosch‐Belmar: data curation, analysis, and visualization. Francesco Paolo Mancuso, Mar Bosch‐Belmar, and Gianluca Sarà: writing the original draft. Francesco Paolo Mancuso, Mar Bosch‐Belmar, Viviana Piermattei, Marco Marcelli, and Gianluca Sarà: review and editing. Viviana Piermattei, Marco Marcelli, and Gianluca Sarà: funding acquisition.

## CONFLICT OF INTEREST STATEMENT

The authors declare no conflicts of interest.

## Supporting information


Appendix S1.

## Data Availability

Data (Mancuso et al., 2026) are available in Dryad at https://doi.org/10.5061/dryad.x69p8czzz.
