# Supplementary material for: Endemic Mediterranean seagrasses poised to survive climate change challenges
Source: Ecol Appl. 2026 Apr 28;36:e70235. doi: 10.1002/eap.70235 (PMC13124460; doi:10.1002/eap.70235)
Supplement: Supplementary file 1 — Appendix S1. [file EAP-36-e70235-s001.pdf]

Appendix S1

Endemic Mediterranean seagrasses poised to survive climate change challenges

Francesco Paolo Mancuso, Mar Bosch-Belmar, Mario Francesco Tantillo, Martina Russi, Viviana Piermattei, Marco Marcelli and Gianluca Sarà

Ecological Applications

Table S1. Pairwise comparisons of seasonal NCP values (Dunn's test with Bonferroni correction).

Comparison	Z	p-value	Sig.
Autumn - Spring	3.25	0.007	**
Autumn - Summer	0.67	1.000	
Spring - Summer	-3.00	0.016	*
Autumn - Winter	2.69	0.042	*
Spring - Winter	-1.06	1.000	
Summer - Winter	2.37	0.106	

* $p < 0.05$, ** $p < 0.01$.

Table S2. Complete overview of all 26 thermal performance models tested from rTPC R package (v. 1.0.2). Models are sorted by fitting success, then by AICc (for fitted models) or alphabetically (for failed models). Green shading indicates the best model (ΔAICc = 0). Yellow shading indicates models excluded due to convergence failure or inadequate fit. AICc = corrected Akaike Information Criterion; ΔAICc = difference from best model; Weight = Akaike weight; df = residual degrees of freedom; σ = residual standard error.

Model	AICc	ΔAICc	weight	df	σ	status	Reference
**Successfully fitted models**							
**Flinn (1991)**	**505.894**	**0.000**	**0.305**	**34**	**207.420**	**Fitted**	**Flinn (1991)**
Gaussian (1987)	506.960	1.066	0.179	34	210.431	Fitted	Lynch & Gabriel (1987)
Quadratic (2008)	508.416	2.522	0.086	34	214.611	Fitted	Montagnes et al. (2008)
Pawar (2018)	508.449	2.555	0.085	33	210.168	Fitted	Kontopoulos et al. (2018)
O'Neill (1972)	509.599	3.705	0.048	33	213.460	Fitted	O'Neill et al. (1972)
Weibull (1995)	509.679	3.785	0.046	33	213.691	Fitted	Angilletta (2006)
Ratkowsky (1983)	509.704	3.810	0.045	33	213.762	Fitted	Ratkowsky et al. (1983)
LRF (1991)	510.103	4.209	0.037	33	214.920	Fitted	Rosso et al. (1993)
Thomas (2012)	510.216	4.322	0.035	33	215.248	Fitted	Thomas et al. (2012)
Rezende (2019)	510.633	4.739	0.029	33	216.463	Fitted	Rezende & Bozinovic (2019)
Spain (1982)	510.899	5.005	0.025	33	217.243	Fitted	Spain (1982)
Briere2 (1999)	510.917	5.023	0.025	33	217.296	Fitted	Brière et al. (1999)
Boatman (2017)	511.543	5.649	0.018	32	214.090	Fitted	Boatman et al. (2017)
Lactin2 (1995)	511.626	5.732	0.017	33	219.389	Fitted	Lactin et al. (1995)
Beta (2012)	512.479	6.585	0.011	32	216.816	Fitted	Niehaus et al. (2012)
Jöhnk (2008)	513.572	7.678	0.007	32	220.042	Fitted	Jöhnk et al. (2008)
Thomas (2017)	517.877	11.983	0.001	32	233.222	Fitted	Thomas et al. (2017)
Hinshelwood (1947)	554.035	48.141	0.000	33	389.137	Fitted	Hinshelwood (1946)

Model	AICc	ΔAICc	weight	df	σ	status	Reference
**Failed Models**							
DeLong (2017)	*NA*	*NA*	*NA*	*NA*	*NA*	*Convergence failure*	DeLong et al. (2017)
Deutsch (2008)	*NA*	*NA*	*NA*	*NA*	*NA*	*Convergence failure*	Deutsch et al. (2008)
Johnson-Lewin (1946)	*NA*	*NA*	*NA*	*NA*	*NA*	*Convergence failure*	Johnson & Lewin (1946)
Kamykowski (1985)	*NA*	*NA*	*NA*	*NA*	*NA*	*Convergence failure*	Kamykowski (1985)
Modified Gaussian (2006)	*NA*	*NA*	*NA*	*NA*	*NA*	*Inadequate fit*	Angilletta (2006)
Sharpe-Schoolfield Full (1981)	*NA*	*NA*	*NA*	*NA*	*NA*	*Convergence failure*	Schoolfield et al. (1981)
Sharpe-Schoolfield High (1981)	*NA*	*NA*	*NA*	*NA*	*NA*	*Convergence failure*	Schoolfield et al. (1981)
Sharpe-Schoolfield Low (1981)	*NA*	*NA*	*NA*	*NA*	*NA*	*Convergence failure*	Schoolfield et al. (1981)

Table S3. Equation, coefficients and thermal performance parameters obtained from the best model (Flinn) for the daily Net Community Production (*NCP*) of *P. oceanica* habitat. T_{opt} = optimum temperature; μ_{max} = maximum rate; T_{br-opt} = optimal thermal breadth; T_{fr}= functional thermal range; CT_{min} = critical thermal minimum; CT_{max} = critical thermal maximum; eh = deactivation energy; Q10 =Q10 value; e = activation energy.

**Model performance parameters**	**value**
T_{opt}	23.083
μ_{max}	540.458
T_{br-opt} (69%)	3.817
T_{fr}	9.925
CT_{min}	11.069
CT_{max}	35.097
Thermal Safety Margin	12.014
Thermal Tolerance	24.028
Skewness	2.031
**Model coefficients**	
eh	0.718
Q_{10}	NA
e	2.749

Note:

Flinn model equation:

$$rate = \frac{1}{1 + a + b\ x\ temp + c\ x\ temp^2}$$

where a= -0.8639539, b = -0.0116270, and c = 0.0002518.

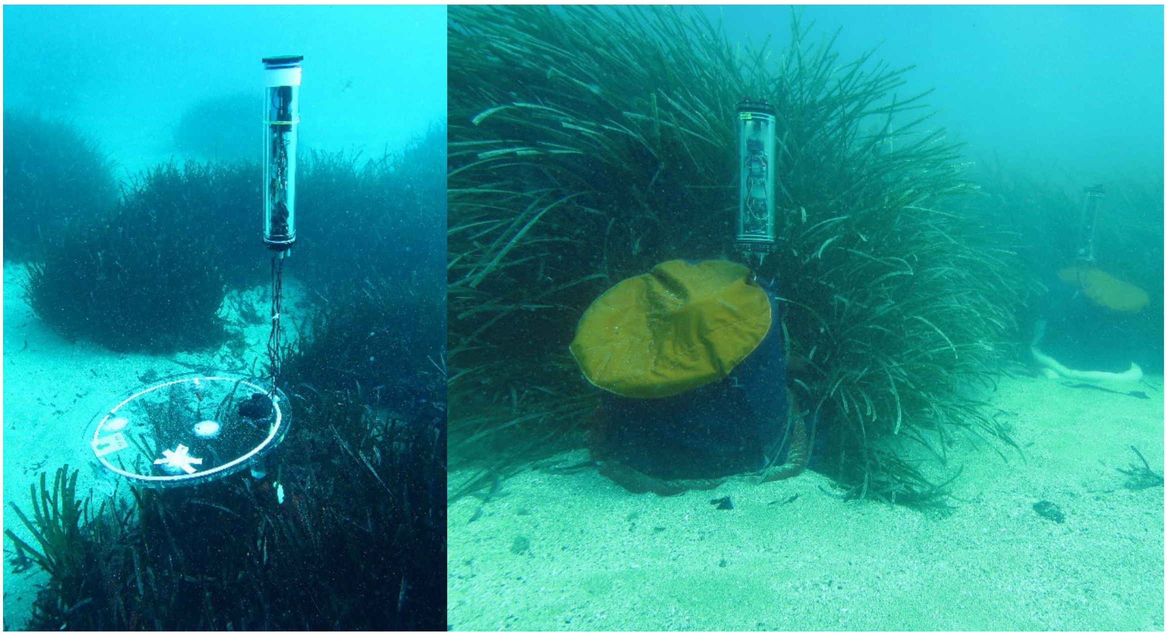

Figure S1. Example of *in situ* benthic chamber and sensoring datalogger measuring *Posidonia oceanica* habitat functioning under light (left) and dark (right) conditions. Images by Francesco Paolo Mancuso.

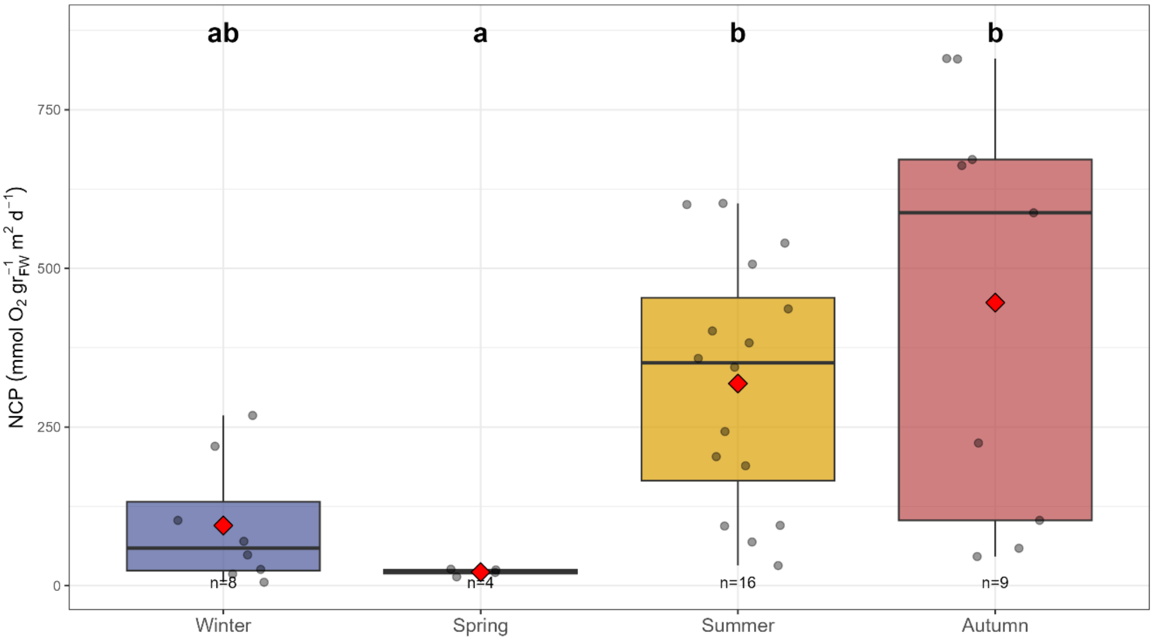

Figure S2. Seasonal field variation in Net Community Production (NCP, mmol O_2 g^{-1}_{FW} m^{-2} d^{-1}) of *Posidonia oceanica* meadows.

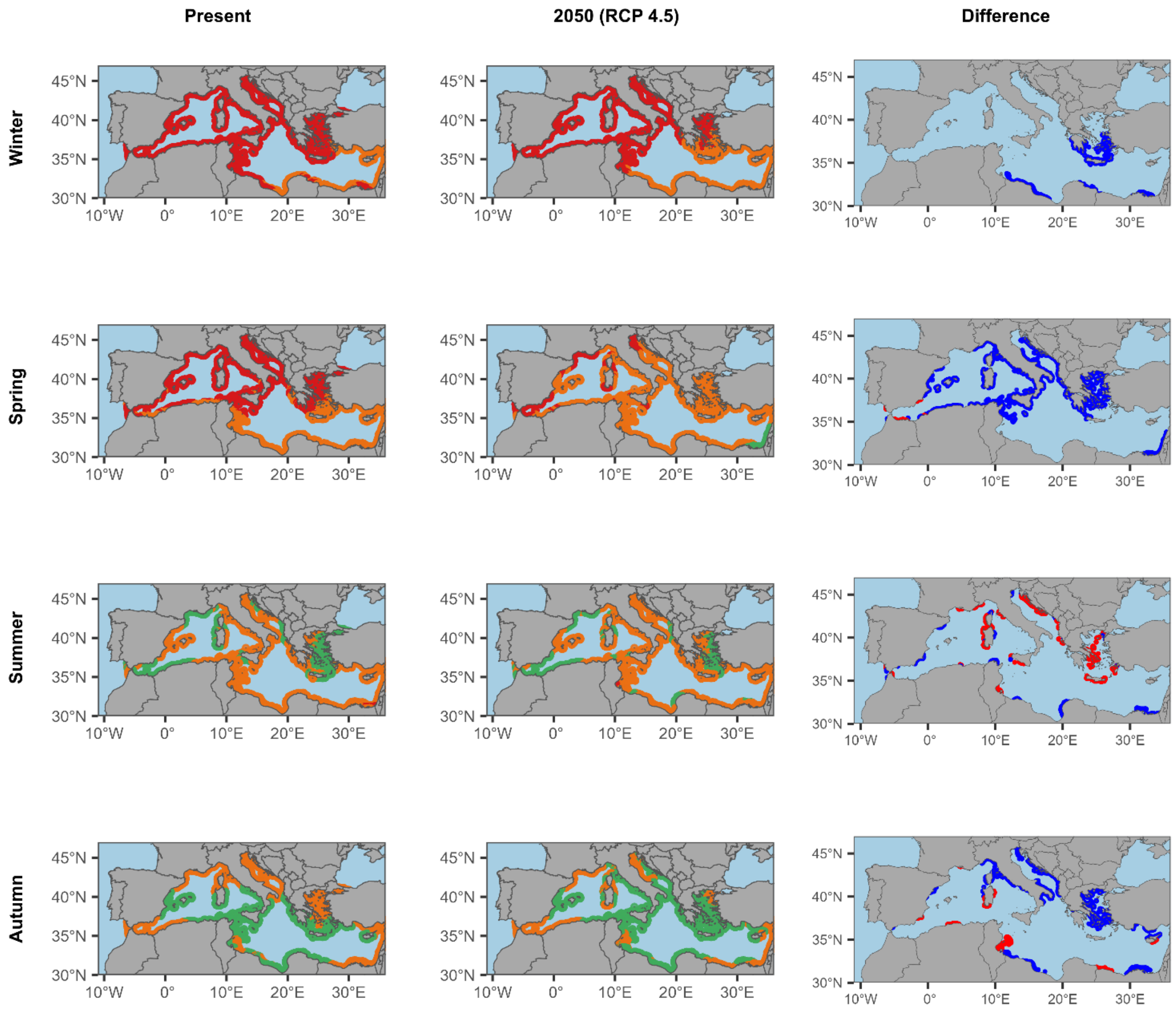

Figure S3. Spatial-temporal present (left column) and future (central column) predictions of the metabolic performance (NCP) of *P. oceanica* habitat along the Mediterranean coasts based on its fundamental thermal niche. Future prediction based on the **4.5 scenario from RCP 2050**. The right column shows differences between future and present predictions. In this column, red color indicates a decrease in the metabolic performance of the *Posidonia* habitat, while blue color highlights an increase in metabolic performance.

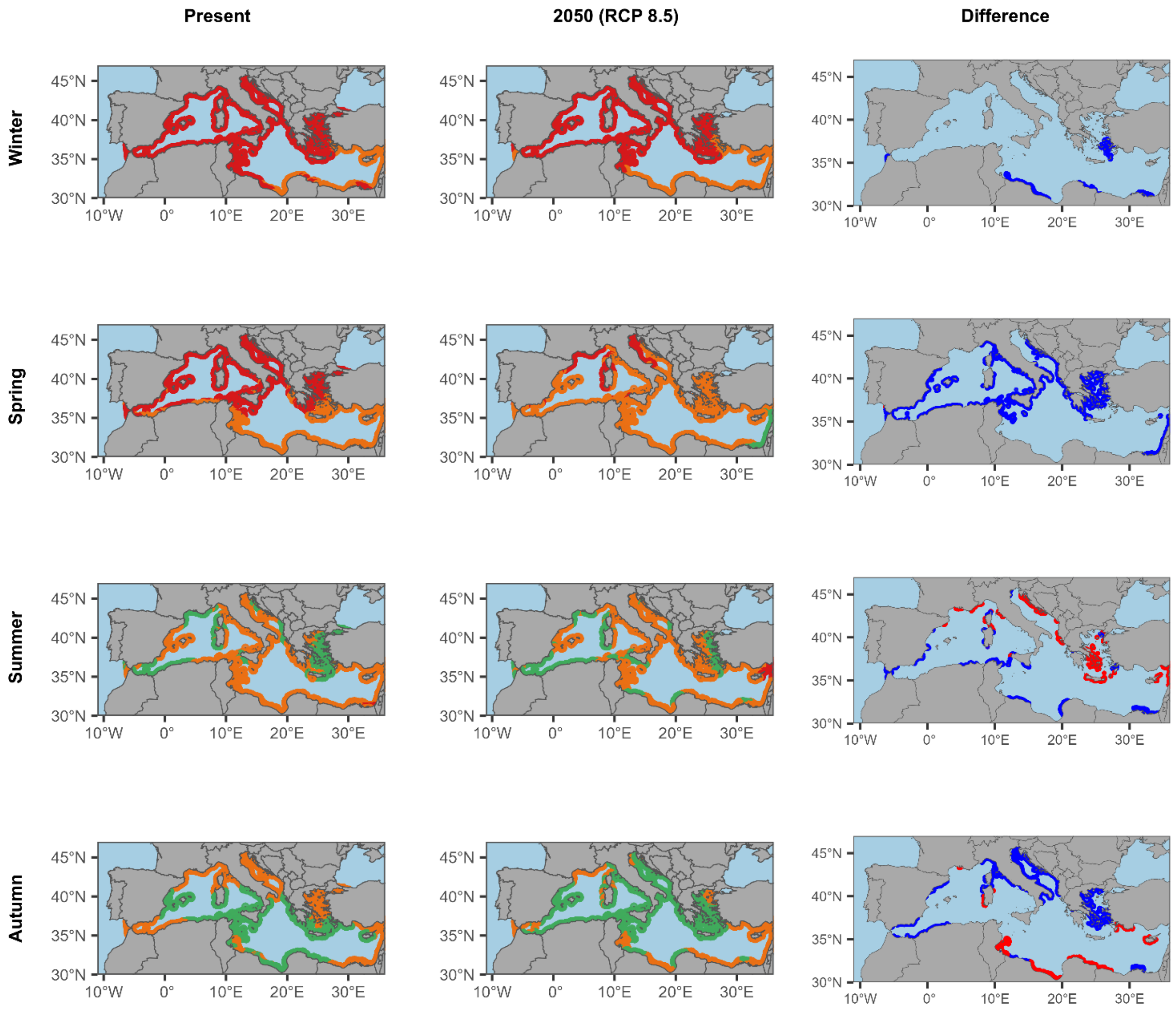

Figure S4. Spatial-temporal present (left column) and future (central column) predictions of the metabolic performance (NCP) of *P. oceanica* habitat along the Mediterranean coasts based on its fundamental thermal niche. Future prediction based on the **8.5 scenario from RCP 2050**. The right column shows differences between future and present predictions. In this column, red color indicates a decrease in the metabolic performance of the *Posidonia* habitat, while blue color highlights an increase in metabolic performance.

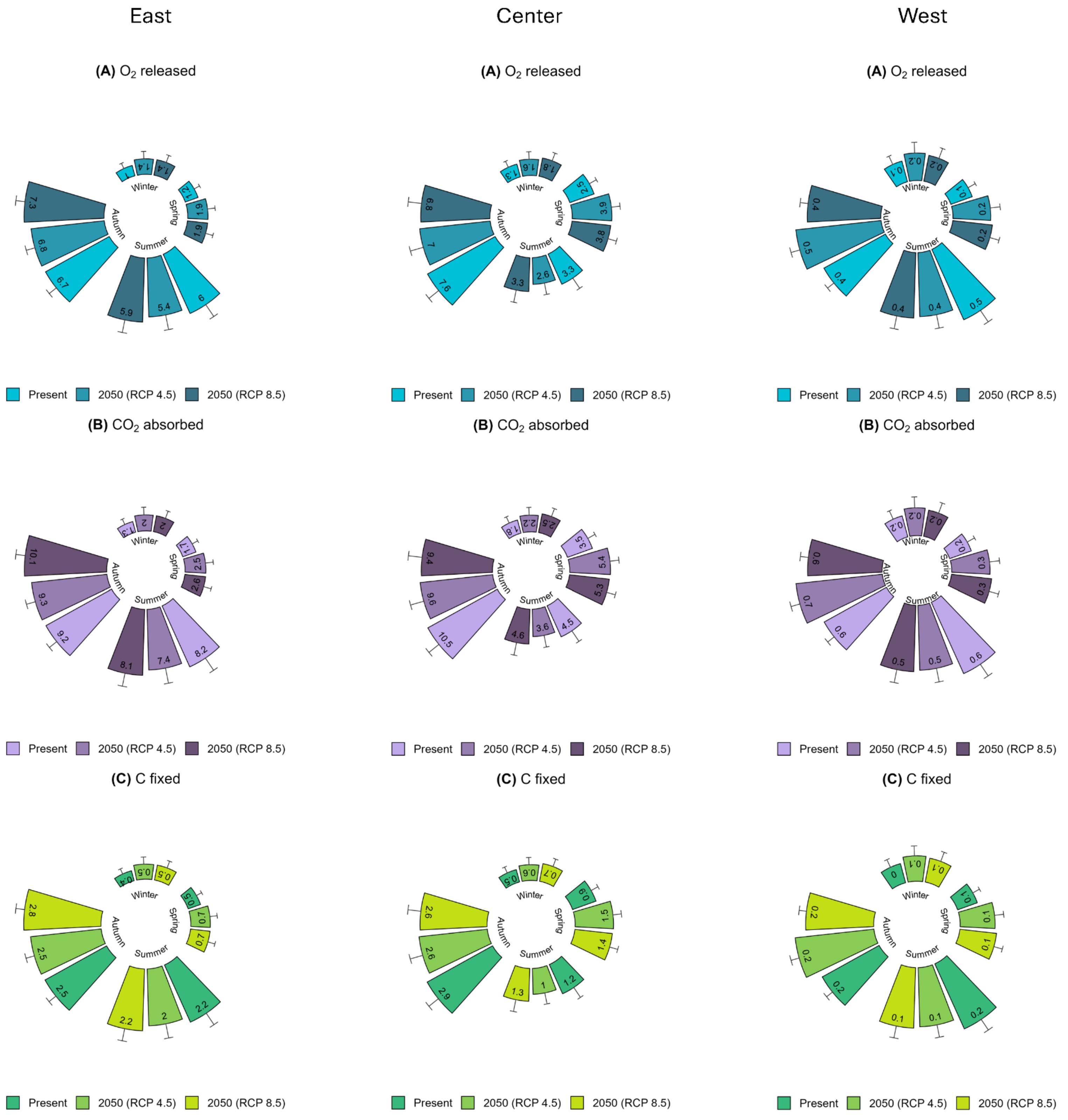

Figure S5. Total million tonnes of O_2 released (a), CO_2 absorbed (b) and C fixed (c) by *P. oceanica* habitat per season and year for the present and projected 2050 (RCP 4.5 and RCP 8.5) seawater temperatures for the main three Mediterranean sectors (west, center, east). Bars show mean + SE.

References

Angilletta, M. J. (2006). Estimating and comparing thermal performance curves. *Journal of Thermal Biology*, 31(7), 541–545. https://doi.org/10.1016/j.jtherbio.2006.06.002 *(Weibull 1995; Modified Gaussian 2006)*

Boatman, T. G., Lawson, T., & Geider, R. J. (2017). A key marine diazotroph in a changing ocean: The interacting effects of temperature, CO_2 and light on the growth of *Trichodesmium erythraeum* IMS101. *PLoS ONE*, 12(1), e0168796. https://doi.org/10.1371/journal.pone.0168796 *(Boatman 2017)*

Brière, J.-F., Pracros, P., Le Roux, A.-Y., & Pierre, J.-S. (1999). A novel rate model of temperature-dependent development for arthropods. *Environmental Entomology*, 28(1), 22–29. https://doi.org/10.1093/ee/28.1.22 *(Briere2 1999)*

DeLong, J. P., Gibert, J. P., Luhring, T. M., Bachman, G., Reed, B., Neyer, A., & Montooth, K. L. (2017). The combined effects of reactant kinetics and enzyme stability explain the temperature dependence of metabolic rates. *Ecology and Evolution*, 7(11), 3940–3950. https://doi.org/10.1002/ece3.2955 *(DeLong 2017)*

Deutsch, C. A., Tewksbury, J. J., Huey, R. B., Sheldon, K. S., Ghalambor, C. K., Haak, D. C., & Martin, P. R. (2008). Impacts of climate warming on terrestrial ectotherms across latitude. *Proceedings of the National Academy of Sciences*, 105(18), 6668–6672. https://doi.org/10.1073/pnas.0709472105 *(Deutsch 2008)*

Flinn, P. W. (1991). Temperature-dependent functional response of the parasitoid *Cephalonomia waterstoni* (Gahan) (Hymenoptera: Bethylidae) attacking rusty grain beetle larvae (Coleoptera: Cucujidae). *Environmental Entomology*, 20(6), 872–876. https://doi.org/10.1093/ee/20.3.872 *(Flinn 1991)*

Hinshelwood, C. N. (1946). *Chemical kinetics of the bacterial cell*. Oxford University Press. *(Hinshelwood 1947)*

Jöhnk, K. D., Huisman, J., Sharples, J., Sommeijer, B., Visser, P. M., & Stroom, J. M. (2008). Summer heatwaves promote blooms of harmful cyanobacteria. *Global Change Biology*, 14(3), 495–512. https://doi.org/10.1111/j.1365-2486.2007.01510.x *(Jöhnk 2008)*

Johnson, F. H., & Lewin, I. (1946). The growth rate of *E. coli* in relation to temperature, quinine and coenzyme. *Journal of Cellular and Comparative Physiology*, 28(1), 47–75. https://doi.org/10.1002/jcp.1030280104 *(Johnson-Lewin 1946)*

Kamykowski, D. (1985). A survey of protozoan laboratory temperature studies applied to marine dinoflagellate behaviour from a field perspective. *Contributions in Marine Science*, 27, 34–44. *(Kamykowski 1985)*

Kontopoulos, D.-G., García-Carreras, B., Sal, S., Smith, T. P., & Pawar, S. (2018). Use and misuse of temperature normalisation in meta-analyses of thermal responses of biological traits. *PeerJ*, 6, e4363. https://doi.org/10.7717/peerj.4363 *(Pawar 2018)*

Lactin, D. J., Holliday, N. J., Johnson, D. L., & Craigen, R. (1995). Improved rate model of temperature-dependent development by arthropods. *Environmental Entomology*, 24(1), 68–75. https://doi.org/10.1093/ee/24.1.68 *(Lactin2 1995)*

Lynch, M., & Gabriel, W. (1987). Environmental tolerance. *The American Naturalist*, 129(2), 283–303. https://doi.org/10.1086/284635 *(Gaussian 1987)*

Montagnes, D. J. S., Morgan, G., Bissinger, J. E., Atkinson, D., & Weisse, T. (2008). Short-term temperature change may impact freshwater carbon flux: A microbial perspective. *Global Change Biology*, 14(12), 2823–2838. https://doi.org/10.1111/j.1365-2486.2008.01700.x *(Quadratic 2008)*

Niehaus, A. C., Angilletta, M. J., Sears, M. W., Franklin, C. E., & Wilson, R. S. (2012). Predicting the physiological performance of ectotherms in fluctuating thermal environments. *Journal of Experimental Biology*, 215(4), 694–701. https://doi.org/10.1242/jeb.058032 *(Beta 2012)*

O'Neill, R. V., Goldstein, R. A., Shugart, H. H., Mankin, J. B., & Booth, R. S. (1972). *Terrestrial ecosystem energy model, eastern deciduous forest biome* (Memo Report 72-19). International Biological Program. *(O'Neill 1972)*

Ratkowsky, D. A., Lowry, R. K., McMeekin, T. A., Stokes, A. N., & Chandler, R. E. (1983). Model for bacterial culture growth rate throughout the entire biokinetic temperature range. *Journal of Bacteriology*, 154(3), 1222–1226. https://doi.org/10.1128/jb.154.3.1222-1226.1983 *(Ratkowsky 1983)*

Rezende, E. L., & Bozinovic, F. (2019). Thermal performance across levels of biological organization. *Philosophical Transactions of the Royal Society B: Biological Sciences*, 374(1778), 20180549. https://doi.org/10.1098/rstb.2018.0549 *(Rezende 2019)*

Rosso, L., Lobry, J. R., & Flandrois, J. P. (1993). An unexpected correlation between cardinal temperatures of microbial growth highlighted by a new model. *Journal of Theoretical Biology*, 162(4), 447–463. https://doi.org/10.1006/jtbi.1993.1099 *(LRF 1991)*

Schoolfield, R. M., Sharpe, P. J. H., & Magnuson, C. E. (1981). Non-linear regression of biological temperature-dependent rate models based on absolute reaction-rate theory. *Journal of Theoretical Biology*, 88(4), 719–731. https://doi.org/10.1016/0022-5193(81)90246-0 *(Sharpe-Schoolfield Full/High/Low 1981)*

Spain, J. D. (1982). *BASIC microcomputer models in biology*. Addison-Wesley. *(Spain 1982)*

Thomas, M. K., Kremer, C. T., Klausmeier, C. A., & Litchman, E. (2012). A global pattern of thermal adaptation in marine phytoplankton. *Science*, 338(6110), 1085–1088. https://doi.org/10.1126/science.1224836 *(Thomas 2012)*

Thomas, M. K., Aranguren-Gassis, M., Kremer, C. T., Gould, M. R., Anderson, K., Klausmeier, C. A., & Litchman, E. (2017). Temperature–nutrient interactions exacerbate sensitivity to warming in phytoplankton. *Global Change Biology*, 23(8), 3269–3280. https://doi.org/10.1111/gcb.13641 *(Thomas 2017)*
